# Differential Effects of Hearing Loss Mutations in Homomeric P2X2 and Heteromeric P2X2/3 Receptors

**DOI:** 10.3390/cells14070510

**Published:** 2025-03-29

**Authors:** Paula-Luise Wand, Xenia Brünings, Debanjan Tewari, Stefanie Reuter, Ralf Mrowka, Klaus Benndorf, Thomas Zimmer, Christian Sattler

**Affiliations:** 1 Institute of Physiology II, Jena University Hospital, Friedrich Schiller University Jena, 07743 Jena, Thüringen, Germany; paula-luise.wand@uni-jena.de (P.-L.W.); xenia.bruenings@med.uni-jena.de (X.B.); debanjan.tewari@med.uni-jena.de (D.T.); klaus.benndorf@med.uni-jena.de (K.B.); thomas.zimmer@med.uni-jena.de (T.Z.); 2 Experimentelle Nephrologie, KIM III, Universitätsklinikum Jena, Nonnenplan 4, 07743 Jena, Thüringen, Germany; stefanie.reuter@med.uni-jena.de (S.R.); ralf.mrowka@med.uni-jena.de (R.M.); 3 Thimedop, ThIMEDOP—Thüringer Innovationszentrum für Medizintechnik-Lösungen, Nonnenplan 4, UKJ, 07743 Jena, Thüringen, Germany

**Keywords:** P2X receptors, hearing loss mutations, fluorescent ATP, ligand-binding assay, patch-clamp, heteromeric ion channels

## Abstract

P2X receptors are unspecific cation channels activated by ATP. They are expressed in various tissues and found in neuronal and immune cells. In mammals, seven subunits are described, which can assemble into homomeric and heteromeric trimers. P2X2 receptors play important roles in cochlear adaptation to elevated sound levels. Three mutations causing inherited progressive hearing loss have been identified. These mutations localize to the transmembrane domain 1 (V60L), the transmembrane domain 2 (G353R) and a β-sheet linking the ATP binding site to the pore (D273Y). Herein, mutations were studied in human homomeric P2X2 as well as in heteromeric P2X2/3 receptors. We measured their binding of a fluorescently labeled ATP derivative (fATP) and characterized the constructs using the patch-clamp technique. The conclusions from our results are as follows: 1. The mutations V60L and G353R show robust localization on the plasma membrane and binding of fATP, whereas the mutant D273Y has no binding to fATP. 2. The mutation V60L has an increased affinity to fATP compared with the wildtype. 3. The expression of hP2X2 V60L channels reduces cell viability, which may support its role in the pathogenesis of hearing loss. 4. All mutant P2X2 subunits can assemble into P2X2/3 heteromeric channels with distinct phenotypes.

## 1. Introduction

**Figure 1:**
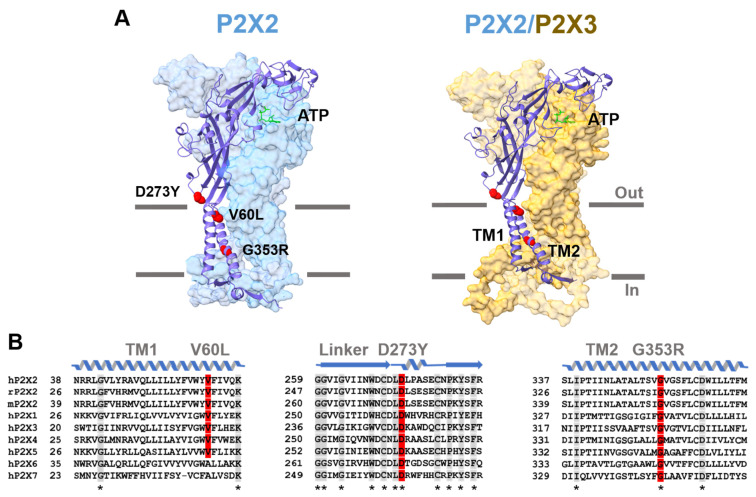
Structures of P2X receptors and hearing loss mutations. (**A**) Structure of a homomeric hP2X2 receptor and a heteromeric hP2X2/3 receptor containing one P2X2 and two P2X3 subunits in the background. One monomer with the three locations (red) of the mutations associated with sensorineural hearing loss is highlighted in front of the trimeric receptor. The structures were generated using alphafold3 and illustrated using chimera [7,11]. One ATP molecule (green) is shown in the binding pocket located between two subunits. The intracellular N- and C-termini are shortened for clarity. (**B**) Sequence alignment containing the 7 human P2X subunits and the P2X2 subunits from rats and mice. The sequences of the transmembrane domain 1 (TM1), a linker coupling the extracellular domain and the pore and transmembrane domain 2 (TM2) are shown. The sequences above are images of the secondary structures, illustrating a helix and a beta sheet (blue arrows). The locations of the mutations are highlighted in red. Conserved amino acids are in the grey background and labeled with an asterisk below the sequences.

A hallmark of the sense of hearing is its remarkably high dynamic range. The mechanisms enabling the cochlea to transduce high sound intensities without damage and to ensure precision in frequency discrimination require several adaptation processes. The purinergic system is involved in the development, function and protection of the cochlea [1]. P2X receptors (P2XRs) belong to this system and are ion channels permeable for cations like sodium, potassium and calcium. They are expressed in various tissues and found in neuronal and immune cells. Thus, the functions of the receptors are also diverse and range from synaptic transmission to pain and inflammation [2,3]. In mammals, seven subunit isoforms are known, which can assemble into homo- and heteromeric trimers. The shape of a single subunit resembles that of a dolphin with a huge extracellular body containing the binding site for ATP and two TM helices forming the fluke with intracellular N- and C-termini [4,5,6]. P2X2 subunits can assemble in homomeric P2X2 receptors and, together with P2X3 subunits, in heteromeric P2X2/3 receptors. Structures were generated by using alphafold3 and are shown in Figure 1A [7]. The binding sites for ATP are located between two subunits and studies suggest that two activated subunits are sufficient to activate the channel [8,9,10].

P2X2 receptors are expressed in the hair and supporting cells of the organ of Corti as well as in the epithelial cells of Reissner’s membrane [12,13]. They are responsible for regulating auditory processes in the inner ear, including sound transmission, mechanotransduction, the electromotility of outer hair cells, gap junction coupling and the recycling of potassium ions. Therefore, they play an important role in noise-induced otoprotection [12,14,15,16]. The concentration of ATP in the endolymph rises from basal levels of around 10 nM due to intense noise exposure and hypoxia to higher levels [17,18]. Consequently, P2X2 receptors become activated and generate a nonspecific cation influx [2,19]. Through the ion efflux from the scala media, including potassium, the endocochlear potential decreases, leading to diminished auditory signal transduction and synaptic transmission [12,20,21]. It is the driving force for signal transduction in the cochlea. The auditory sensitivity is reduced by this adaptation, preventing overstimulation [22]. This P2X2-dependent cochlear mechanism protects the auditory system against noise-induced damage. Mutations in this gene can cause autosomal dominant progressive hearing loss (DFNA41) that is also sensitive to noise exposure [23,24]. Three mutations (V60L, G353R and D273Y) that lead to this kind of sensorineural hearing loss have been identified [23,24,25,26]. V60L localizes to the external end of transmembrane domain 1 (Figure 1A,B) and is in close contact with the pore-forming transmembrane domain 2. Two studies reported a loss of function through the lack of forming functional channels on the plasma membrane [23,27]. Another study reported an uncoupling of the pore and the ATP binding site, resulting in channels with an intrinsic open probability and inward rectification [28]. D273Y localizes to a linker between the external part of the receptor containing the binding sites for ATP and the pore. This region is characterized by a high degree of conservation among the subtypes (Figure 1B). The reported loss of function was explained by the inability of trafficking to the plasma membrane [28]. G353R lies in the pore-forming TM2 and is probably part of a hinge involved in closed–open transitions [29,30,31]. One study showed that these channels are nonfunctional [27], whereas another study reported G353R channels with reduced ATP sensitivity and altered ion selectivity [28]. All reported studies on the mutations used a standard external sodium-rich solution and, thus, sodium was the main permeating ion at negative voltages. An important goal of our work was to extend these studies using a potassium-rich solution, which reflects conditions of the endolymph in the inner ear.

During the neonatal and early postnatal period, P2X3 receptor subunits are transiently expressed in the developing cochlea [32]. It is reported that there are species-specific differences in its expression. In mice, P2X3 is localized in spiral ganglion neurons (SGNs) as well as in inner and outer hair cells. In the inner ear of rats, it is only present in SGNs. After the onset of hearing, there is no detection of P2X3 expression in the inner ear of both species [32,33]. The expression pattern coincides with cochlear synaptic reorganization. This suggests that P2X3-mediated purinergic signaling contributes to developmental processes such as synaptic determination and the establishment of functional neurotransmission [33]. The overlapping time-dependent expression of P2X2 and P2X3 subunits in different cells of the inner ear enables the formation of heteromeric P2X2/3 channels in which the mutations can alter their functions [34,35,36,37,38].

Herein, we studied the binding of three mutant P2X2 receptors using a fluorescent ATP derivative as well as their activation with potassium as the permeating ion. We further tested the ability of forming heteromeric P2X2/3 channels containing mutant P2X2 subunits and wildtype P2X3 subunits. Our work demonstrates that the three mutations impair P2X2 channel function in different ways and that all mutant P2X2 subunits can assemble into P2X2/3 heteromeric channels with distinct phenotypes.

## 2. Materials and Methods

### 2.1. Molecular Biology and Cell Culture

The human P2X2 splice isoform B (hP2X2) and P2X3 (hP2X3) were amplified from cDNA (BD Biosc. 4030043, Heidelberg, Germany) and introduced using respective restriction sites into pcDNA5/FRT/TO. For the co-expression of hP2X2 and hP2X3 subunits, a plasmid containing two expression cassettes with the tetracycline-inducible CMV promoter, the hP2X gene and the BGH termination sequence was created using PCR and unique restriction sites, as previously described [39]. Mutations were introduced using overlapping PCR strategies and unique restriction sites. All plasmids were verified via a restriction analysis and partial sequencing (Microsynth SEQLAB, Göttingen, Germany). The luciferase reporter plasmid was generated by replacing the NF-κB responsive promoter from pCDH-Luc-EF1-Puro with a CMV promoter PCR product flanked by restriction enzyme sites for *XhoI* and *EcoRI* [40].

Human P2X receptors were expressed in HEK cell lines containing an inducible promoter system (Flp-In-T-REx 293, Invitrogen, Waltham, MA, USA). The used cell lines were hP2X2 B (RRID: AC line CVCL_D6U1), hP2X3 (RRID: AC line CVCL_D6U3), hP2X2/3 (RRID: AC line CVCL_D6U2), hP2X2 B V60L (RRID: AC line CVCL_E2Y1), hP2X2 B D273Y (RRID: AC line CVCL_E2Y2), hP2X2 B G353R (RRID: AC line CVCL_E2Y3), h P2RX2(B)V60L + P2RX3 (RRID: AC line CVCL_E2Y4), hP2RX2(B)D273Y + P2RX3 (RRID: AC line CVCL_E2Y5) and h P2RX2(B)G353R + P2RX3 (RRID: AC line CVCL_E2Y6).

Cells were cultured in MEM supplemented with non-essential amino acids (Gibco, Waltham, MA, USA), 10% FCS and antibiotics, according to the manufacturer’s instructions. For stable cell lines, Flp-In-T-REx 293 cells were transfected using the calcium phosphate method with a solution of plasmids (0.3 µg pcDNA5/FRT/TO P2X gene and 1.5 µg pOG44) and selected for hygromycin resistance. Stable clones were cultured until a passage number of approximately 20 was obtained. For electrophysiological measurements, cells were seeded on glass coverslips and used 16–24 h after tetracycline induction (1 µg/µL). For binding measurements, cells were induced for 24 h and plated on chambered coverslips or 96-well plates coated with poly L-lysine 1–3 h before recordings.

### 2.2. Viability Assay

Stable cell lines with inducible P2X genes were plated at 80–90% confluence in a 6-well format with a glow medium. The glow medium consisted of DMEM high glucose without phenol red (Thermo Fisher Scientific, Langenselbold, Germany) supplemented with 10% fetal calf serum, 1% Pen/Strep, 10 mM HEPES and 250 µM Luciferin D. Six hours after the transfection of firefly luciferase using lipofectamin 2000 (Thermo Fisher Scientific, Langenselbold, Germany), the cells were transferred to a 96-well plate (12 wells per subtype). After overnight incubation, the plate was transferred to a top count reader and the expression of the P2X receptors was induced using tetracycline (1 µg/mL). Cells were monitored for 48 h. The 96-well plates were kept at 35 °C in the TopCount NTX luminescence plate reader (Packard, Detroit, MI, USA) in a buffered glow medium. The principle of this viability assay was as follows. The light reaction depended on the substrate and the ATP concentration. The luciferase was produced in the cells and, in the case of healthy living cells, we obtained a high light signal because the enzyme firefly luciferase, the substrate luciferin and ATP were present in the cells. If the cell died, the concentration of ATP dropped and the light signal diminished [41]. Luminescence was determined in the plate reader every 3 min as counts per second (cps). For the analysis, the ratio of the signal after 48 h and the starting value were calculated for each well.

### 2.3. Electrophysiology

Currents were recorded in the whole-cell configuration using a standard patch-clamp technique [42]. The patch pipettes were pulled from borosilicate glass (ID 1.0 mm and OD 2.0 mm; VITROCOM, Mountain Lakes, NJ, USA) using a micropipette puller (P-97, Sutter Instrument, Novato, CA, USA). The pipettes were filled with an intracellular solution containing (mM) 142 KCl, 5 BAPTA, 5 EGTA and 10 HEPES at pH 7.4. The pipette resistance was 1.5 to 6.0 MΩ. The bath solution contained (mM) 142 KCl, 2 CaCl_2_, 1 MgCl_2_, 10 HEPES and 10 glucose at pH 7.4. For control measurements, 142 mM KCl was replaced with 142 mM NaCl in the internal and external solutions. Lifted cells were placed in front of the outlet of the application system with three barrels (inner diameter ~600 µm; Warner Instruments, Hamden, CT, USA) controlled by a step motor (SF-77B, Warner Instruments). Currents were activated by test pulses ranging from −140 to +80 mV in 20 mV increments from a holding potential of 0 mV, with each test pulse lasting 150 ms followed by activation at −60 mV for 100 ms. The time between pulses was 500 ms. Series resistance was compensated for using Patchmaster software up to 80%. At very negative potentials and high expressions of some receptors, a voltage error arose that was not neglectable. The data were not further corrected for this. For ligand jump experiments, 100 µM ATP solutions were applied at a holding potential of −50 mV for one second. The recordings were digitalized using a HEKA EPC 10 (Stuttgard, Germany) amplifier and Patchmaster 2019 software. The sampling rate was 10 kHz and the currents were filtered online at 2.9 kHz using a 4-pole Bessel filter.

### 2.4. Confocal Microscopy

For measurements of ligand binding, cells were incubated with the fluorescent ATP derivative 2-[DY-547P1]-AHT-ATP (fATP, Biolog, Bremen, Germany) in chambered coverslips or a 96-well format. The surface was coated with Poly-L-Lysine and washed twice with PBS. Cells were seeded two hours before measurements. This allowed us to obtain rounded cells providing a higher fluorescent signal. The solution was counterstained with the red fluorescence dye Dy647 (Dyomics, Jena, Germany). An automated algorithm was employed to detect the specific binding of fATP to the receptors on the plasma membrane. Measurements were performed using a solution containing (mM) 142 NaCl, 10 EGTA, 10 HEPES and 10 glucose at pH 7.4 because of the reported higher affinity of fATP to hP2X2 receptors when no complex with divalent ions is formed [39]. The method was previously described in detail [43].

### 2.5. Analysis

Voltage activation relationships from whole-cell measurements of HEK293 cells were obtained from peak currents during test pulses at different ATP concentrations. Time-dependent deactivation was described by fitting the time courses with single exponentials, yielding *t_off_* for deactivation. Data were presented as the mean ± SEM. Statistical significance was tested by using the Student’s *t*-test for unpaired data or the Mann–Whitney U test. The numerical and statistical analyses were carried out using Origin.Lab 2019 and Fitmaster 2019 software. Differences with a *p*-value smaller than 0.05 were considered to be significant.

## 3. Results

### 3.1. Binding of fATP to Wildtype P2X Receptors and Their Activation with Potassium as the Permeating Ion

**Figure 2:**
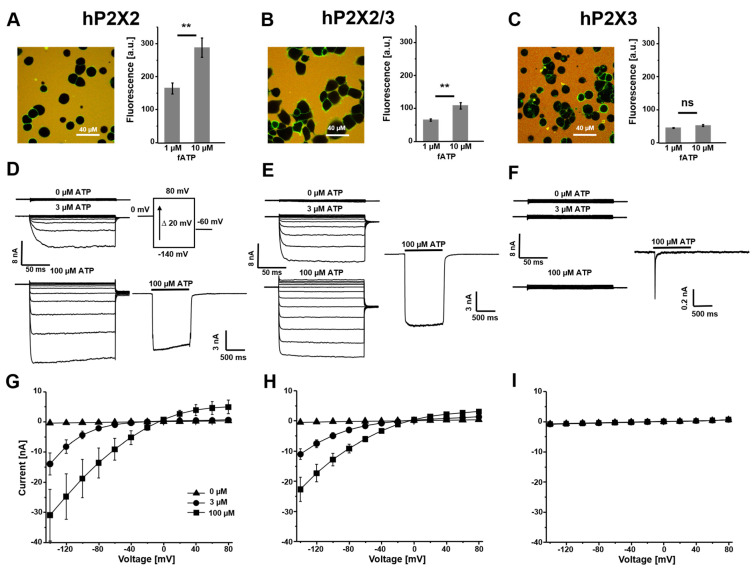
Binding and activation of P2X wildtype receptors. (**A**–**C**) Representative confocal images of HEK 293 cells, stably expressing human P2X receptors, binding fATP at 1 µM (left). Quantification of the specific binding signal at 1 µM and 10 µM fATP. The bar graphs are derived from 15–40 images and represent the mean ± SEM (right). (**D**–**F**) Representative traces of recordings from stably expressing HEK 293 cells in the whole-cell configuration at different voltages in the presence of applicator-applied ATP (left) and ligand activation at a constant holding potential of −50 mV at the given ATP concentration (right). (**G**–**I**) Current–voltage relationships from −140 mV to +80 mV. One data point represents the mean ± SEM from 5–7 cells. ** *p* < 0.01; ns: not significant.

The localization of wildtype (wt) P2X2, P2X3 and P2X2/3 receptors on the plasma membrane expressed from inducible stable HEK293 cells was tested using fATP. In line with previous results [39], there was robust staining with 1 µM fATP (Figure 2A–C) and no staining on control cells.

The ratio of the binding signal at 1 µM and 10 µM fATP was used to estimate the affinity of the receptors. For homomeric hP2X2 and heteromeric hP2X2/3 receptors, a similar ratio (0.57 and 0.54) was obtained, whereas P2X3 showed no difference between these concentrations. This suggested that 1 µM fATP had already had a saturating effect and that hP2X3 receptors had a higher affinity to fATP. These data matched the results of our previous study [39]. The responses to voltage steps showed strong inward currents in potassium containing the bath solution for hP2X2 and hP2X2/3 receptors (Figure 2D,E,G,H). The maximal amplitudes at −140 mV for 100 µM ATP were 31.0 ± 8.6 nA and 22.7 ± 4.0 nA. Because of fast desensitization, typical of hP2X3 receptors [36,44], there was no voltage-activated current with ATP under steady-state conditions (Figure 2F,I). The functional expression was shown under non-equilibrium conditions using fast jumps into a high concentration of ATP at −50 mV. hP2X2 and hP2X2/3 showed characteristic fast activation with little desensitization and hP2X3 receptors showed even faster activation followed by nearly complete desensitization in less than 100 ms. The functional differences between homomeric hP2X2 and heteromeric P2X2/3 receptors were shown in a parallel study with the same cell lines using subtype-specific ligands for binding and activation as well as a Mg^2+^-dependent block of hP2X2 receptors [39].

### 3.2. Homomeric hP2X2 V60L Receptors Show an Increased Affinity to fATP and Cause a Decrease in Cell Viability

**Figure 3:**
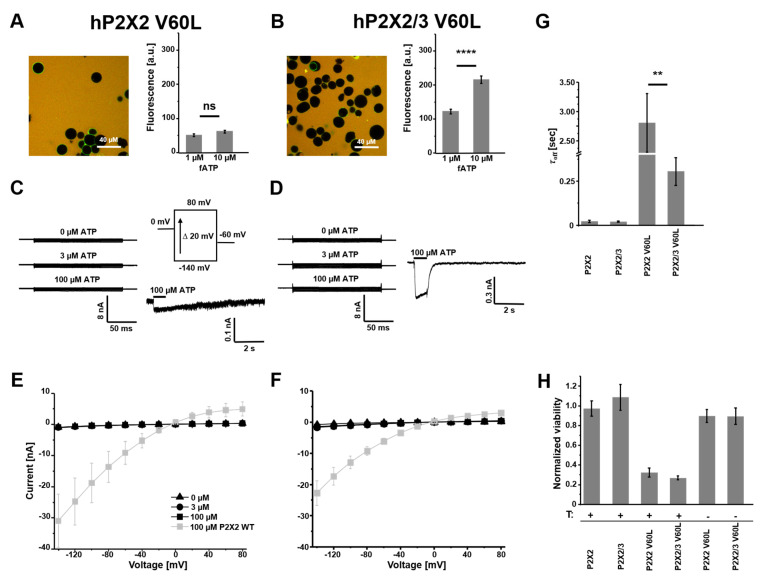
Binding and activation of P2X V60L mutant receptors. (**A**,**B**) Representative confocal images of HEK 293 cells, stably expressing human P2X receptors, binding fATP at 1 µM (left). Quantification of the specific binding signal at 1 µM and 10 µM fATP. Bar graphs are derived from 15–40 images and represent the mean ± SEM (right). (**C**,**D**) Representative traces of recordings from stably expressing HEK 293 cells in the whole-cell configuration at different voltages in the presence of applicator-applied ATP (left) and ligand activation at a constant holding potential of −50 mV at the given ATP concentration (right). (**E**,**F**) Current–voltage relationships from −140 mV to +80 mV. One data point represents the mean ± SEM from 5–9 cells. (**G**) Deactivation time constants derived from exponential fits after removal of 100 µM ATP are shown as the mean ± SEM. (**H**) Viability of the cells was expressed as the normalized luminescence signal from the luciferase reaction after 48 h of tetracycline treatment; 12 wells in a 96-well plate format were analyzed. T: tetracycline treatment. ** *p* < 0.01; **** *p* < 0.0001; ns: not significant.

The binding of fATP to P2X2-V60L-containing receptors demonstrated the functionality of ATP binding sites and the localization of these mutant receptors on the plasma membrane (Figure 3A,B). The binding signal at 1 µM and 10 µM fATP to homomeric P2X2 V60L was not different. Therefore, we concluded that there was a higher affinity to the ligand compared with wildtype P2X2 and heteromeric P2X2/3 V60L receptors. Furthermore, the binding measurements indicated the formation of heteromeric P2X2/3 V60L channels because of the higher affinity of the respective monomeric assemblies, which are also possible in co-expressions.

During the experiments, we observed a higher loss of cells expressing the P2X2 V60L subunit compared with all other examined cells (Figure 3A). Therefore, the time between the induction of expression with tetracycline and the measurements was reduced to 12–16 h. Additionally, we quantified the viability of the cell lines using a co-expression of firefly luciferase in a glow medium containing Luciferin D. Healthy cells produce ATP and, therefore, provide a substrate for the luciferase reaction. Normalized luminescence, obtained from cells expressing wt P2X2 and P2X2/3 receptors as well as from the cell lines P2X2 V60L and P2X2/3 V60L without an induction of expression, showed a stable signal after 48 h culture in the luminescence counter (Figure 3H). Both P2X2 V60L and P2X2/3 V60L cell lines with an induction of expression showed a decrease in the luminescence signal 48 h after induction. There was no difference between P2X2 V60L and P2X2/3 V60L cells (0.32 ± 0.05 vs. 0.27 ± 0.02, n = 12 and *p* = 0.36), indicating a dominant effect of the mutant subunit. Electrophysiological characterization using the voltage-step protocol showed no ATP effect on the amplitudes at −140 mV (977.1 ± 125.7 pA vs. 957.8 ± 142.6 pA; n = 8). Furthermore, we could not observe any increase in current at negative voltages (Figure 3C,D). Figure 3E,F summarize the voltage-triggered activation of the V60L-containing receptors compared with the respective wt receptors (grey). The discrepancy between the robust binding of fATP and the absence of ATP-modulated voltage activations led us to perform ligand jump experiments at a constant holding potential of −50 mV. The rapid application of 100 µM ATP resulted in fast activation and little desensitizing currents (Figure 3C,D) but with very small amplitudes compared with the wt receptors. The removal of ATP resulted in slow deactivation compared with the wt receptors and was even slower for homomeric P2X2 V60L receptors. The obtained τ_off_ values are summarized in Figure 3G and suggest, in addition to the binding measurements, an effect on binding and gating when V60L was present in the channels.

### 3.3. hP2X2 D273Y Subunits Do Not Form Functional Homomers but Can Assemble into P2X2/3 D273Y Heteromers

**Figure 4:**
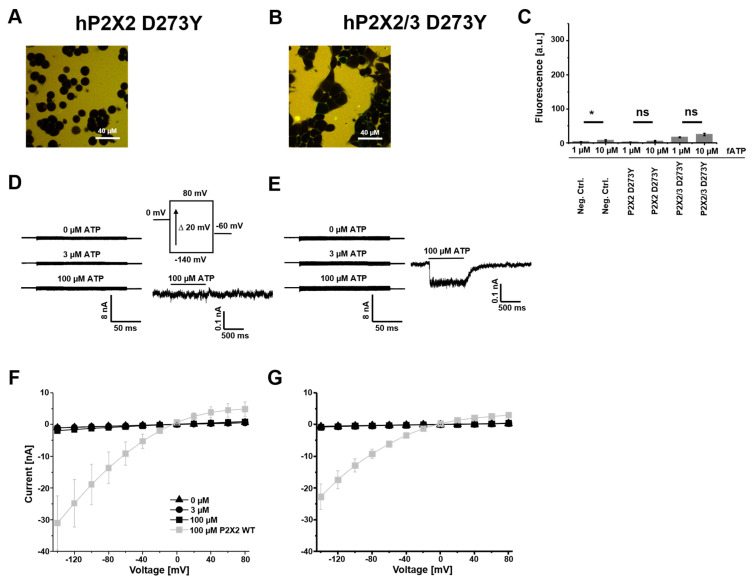
Binding and gating of P2X D273Y mutant receptors. (**A**,**B**) Representative confocal images of HEK 293 cells, stably expressing human P2X receptors, binding fATP at 1 µM. (**C**) Quantification of the specific binding signal at 1 µM and 10 µM fATP. The bar graphs are derived from 15–40 images and represent the mean ± SEM (right). (**D**,**E**) Representative traces of recordings from stably expressing HEK cells in the whole-cell configuration at different voltages in the presence of applicator-applied ATP (left) and ligand activation at a constant holding potential of −50 mV at the given ATP concentration (right). (**F**,**G**) Current–voltage relationships from −140 mV to +80 mV. One data point represents the mean ± SEM from 5–13 cells. * *p* < 0.05; ns: not significant.

Previously, it was shown that P2X2 D273Y subunits fail to traffic to the plasma membrane, forming functional channels [28]. We confirmed these results under our conditions by using fATP as a reporter for ATP binding sites on the plasma membrane (Figure 4A,C). Furthermore, we failed to detect currents in response to voltage and ligand applications for homomeric receptors (Figure 4D,F). The co-expression of mutant P2X2 subunits together with wt P2X3 subunits resulted in a small binding signal using fATP (Figure 4B,C).

The current–voltage relationships showed no ATP modulation and inward rectification (Figure 4E,G). The loss of function is obvious when comparing the absolute current values with the respective wt receptors (grey) in Figure 4F,G. The fast application of 100 µM ATP at −50 mV generated a tiny non-desensitizing current in 6 out of 13 cells (Figure 4E). This new phenotype showed the assembly of P2X2/3 D273Y heteromers and a suppression of fast desensitizing P2X3 receptors in the presence of mutant P2X2 D273Y subunits.

### 3.4. Homomeric hP2X2 G353R Receptors Show Reduced Function with Potassium as the Permeating Ion

**Figure 5:**
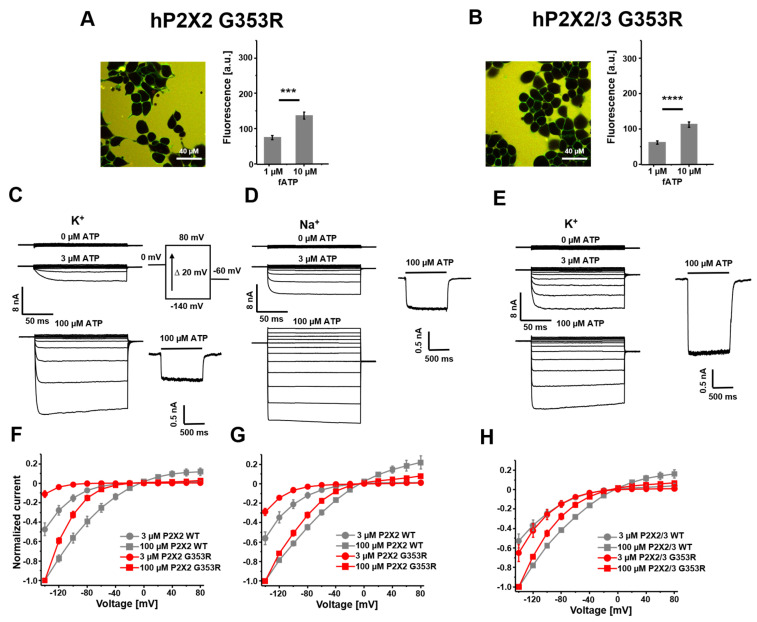
Binding and activation of P2X G353R mutant receptors. (**A**,**B**) Representative confocal images of HEK 293 cells, stably expressing human P2X receptors, binding fATP at 1 µM (left). Quantification of the specific binding signal at 1 µM and 10 µM fATP. The bar graphs are derived from 15–40 images and represent the mean ± SEM (right). (**C**–**E**) Representative traces of recordings from stably expressing HEK 293 cells in the whole-cell configuration at different voltages in the presence of applicator-applied ATP (left) and ligand activation at a constant holding potential of −50 mV at the given ATP concentration (right). (**F**–**H**) Normalized current–voltage relationships from −140 mV to +80 mV. One data point represents the mean ± SEM from 5–9 cells. The maximum amplitude of the current signals was normalized with respect to the maximum current amplitude of 100 µM ATP at −140 mV. *** *p* < 0.001; **** *p* < 0.0001.

P2X2 G353R subunits trafficked to the plasma membrane and formed homomeric P2X2 G353R and heteromeric P2X2/3 G353R channels with functional binding sites for fATP (Figure 5A,B). The ratios of the binding signal at 1 µM and 10 µM fATP were 0.55 and 0.55, which were close to the ratios of the respective wt receptors (0.57 and 0.60). This suggests a lack of relevant effects of the mutation on ATP binding sites.

In response to voltage pulses, we found strong inward currents that were positively modulated with ATP for homomeric P2X2 G353R and heteromeric P2X2/3 G353R channels (Figure 5C–E). The amplitudes at −140 mV and 100 µM ATP for the mutants were in the range of those for the respective wt channels (18.5 ± 2.8 nA for P2X2 G353R, n = 8, and 14.1 ± 2.1 nA for P2X2/3 G353R, n = 9). However, we found lower current amplitudes at 3 µM ATP for the mutant compared with the wildtype (Figure 5C,D,F,G). This reduced sensitivity for ATP was previously reported under conditions with sodium as the main permeating ion and is further reduced if potassium is the permeating ion [28]. We normalized the current–voltage relationships to maximal currents at −140 mV and 100 µM ATP for a better comparison of their shapes and relative amplitudes (Figure 5F–H). In addition to a changed sensitivity, we confirmed steeper current–voltage relationships for the homomeric mutant channels. Furthermore, we observed reduced outward currents compared with the wt receptors. This effect was even stronger in a potassium-rich solution, supporting the findings about altered ion selectivity in the context of the G353R mutant [28]. The co-expression of mutant P2X2 G353R subunits and wt P2X3 subunits resulted in a new phenotype with unchanged ATP sensitivity compared with the respective wt heteromeric channels and only slightly steeper current–voltage relationships (Figure 5H).

## 4. Discussion

Hearing loss is the most common sensory deficit and its prevalence is increasing. Insufficient knowledge about pathomechanisms and a lack of any specific pharmacotherapy calls for research on underlying molecular mechanisms. The purinergic system is involved in cochlear (patho)physiology and ATP-gated P2X receptors are important players in it. Herein, we studied P2X2 mutants (V60L, D273Y and G353R) related to dominantly progressive sensorineural hearing loss to shed some light on this intricacy. Extending previous studies with partly conflicting results [23,27,28], we used a fluorescent ATP derivative to report receptor localization and ligand binding as well as patch-clamp recordings in a potassium-rich external solution and co-expression with wt P2X3 subunits to study P2X2/3 heterotrimerization.

Previously, it was reported that P2X2-V60L-transfected cells show no [23] or almost no [27] ATP-gated current or that the receptors are insensitive to ATP and are constitutively active, probably because of an uncoupling of ATP binding and pore opening [28]. We could not detect these characteristics in our findings. We observed robust binding to fATP, but no channels with an intrinsic open probability. Upon ATP application, we measured currents, although they were minimal compared with the wt P2X2 receptor. In addition, we noticed reduced cell viability after the induction of expression. We can only speculate about the discrepancies between our results and previous studies. It might be that there is some kind of selection in the electrophysiological measurements. Highly expressing cells were dying and unused in the recordings, but the staining of dying cells with fATP was possible. The surviving cells in our recordings were probably cells with only a few channels. The heteromeric P2X2/3 channels with mutated P2X2 V60L subunits also caused cell death. Staining with fATP was possible but, again, only tiny ATP-triggered currents could be observed. Thus, heteromeric channels were formed but with a dominant effect of the mutation. P2X2 receptors are expressed in inner and outer hair cells, supporting cells, spiral ganglion neurons and epithelial cells of Reissner’s membrane [13,20,45]. It is plausible that these cells are damaged and dying if they express the V60L mutant. That would directly affect sound transduction, auditory transmission and ion homeostasis, including potassium recycling.

Although TM1 is not part of the pore in P2X receptors, strong effects of mutations on permeation, sensitivity to ATP and current time courses have been reported [46,47,48]. The coupling of TM1 via V48 (corresponding with V60 in human P2X2) and TM2 via I328 was shown in rat P2X2 channels by crosslinking introduced cysteines at these positions [47]. The slowed deactivation of V60L receptors in our study (Figure 3G) may have been caused by slower closing transitions and the slower unbinding of ATP due to higher affinity to the receptor. We could show, for homomeric P2X2 V60L receptors, a higher affinity for direct binding measurements using fATP. However, in heteromeric channels containing one or two copies of P2X2 V60L, the binding was similar to the wt heteromeric P2X2/3 channels. This suggests an interdependence of binding and gating, causing the V60L phenotypes.

Homomeric P2X2 receptors assembled from mutant D273Y subunits had no detectable binding to fATP. Consequently, no ATP-mediated currents were detectable. This matched previous investigations of this receptor, proposing that the receptor fails to traffic to the plasma membrane [28]. However, a co-expression with wt P2X3 subunits could restore some of the impaired receptor functions by heteromerization. There was binding to fATP and small ATP-activated currents could be detected. Taken together, this mutation causes a loss of function; even in heteromeric channels, the mutant dominated with a suppression of the current.

Mutant G353R revealed significantly diminished currents at an ATP concentration of 3 µM, especially in a potassium solution. Previous studies reported a maintained considerable conductance but alterations in ion selectivity, consistent with its location in the pore-lining TM2 helix [28]. Our measurements demonstrated the importance of this altered ion selectivity regarding the potassium-rich composition of the endolymph. The G353R mutation is the only one that could restore the function of the receptor by heteromerization. This is in agreement with previous co-transfection experiments of mutations with wildtype P2X2 subunits [27].

The three mutations leading to sensorineural hearing loss (DFNA41) exhibited different functional impairments, including higher affinity (V60L), no binding of ATP (D273Y) and an altered ion selectivity (G353R). This highlights the importance of the otoprotection of P2X2 signaling in the cochlea. As the P2X2 receptor is expressed in the supporting cells [49], purinergic signaling contributes to ion homeostasis and the recycling of potassium ions and, therefore, influences the endocochlear potential [14]. During noise exposure, the ATP concentration in the endolymphatic compartment rises [18], resulting in activated P2X2 receptors. Consequently, there is a cation influx [50] in cells expressing P2X2 receptors and having contact with the endolymph, like Hensen’s, Deiters’, Claudius’ and outer sulcus cells [13,50]. Thus, the endocochlear potential, known as the driving force for auditory transduction [51], decreases and this ATP-induced mechanism provides an adaptation and cochlear protection against overstimulation during noise exposure. The described cation shunt is part of the potassium recycling in the supporting cells of the stria vascularis [14].

P2X2 receptors are ubiquitously expressed in various tissues beyond the cochlea. They play modulating roles in stress, inflammatory reactions and immune responses and take part in sensory information transmission, including taste and pain [52,53,54]. Possibly, the mutations in the P2X2 gene could also affect other physiological functions in addition to the auditory system. A conditional knock-out of P2X2 receptors in pyramidal neurons showed resilience of chronic stress-induced depression-like behaviors [55]. There is good analgesic efficacy in preclinical studies using P2X2/3 inhibitors [56]. Previous studies found diminished responses to tastants [57] and mild vestibular dysfunction in knock-out mice [58]. Consequently, impaired P2X2 functions due to mutations could also have an impact similar to inhibitors or receptor knock-out models. It was shown that mice expressing V60L had pain hypersensitivity, correlating with our assumption of the higher affinity of the V60L mutant, and vestibular dysfunction [49]. This also suggests male fertility problems because P2X2 receptors are expressed in mouse spermatozoa and it was not possible to pair male knock-in mice [49,59].

From a biophysical point of view, it might be interesting to study the mutations in heteromeric receptors with defined copies of mutant subunits together with wt P2X2 or P2X3 subunits using subunit concatenation. Previously, it was shown that P2X concatemers are functional [9,10,60]. Furthermore, concatemers containing disease-related mutations or disabled binding sites have been extensively studied [61,62,63] and have provided fruitful information on the function of individual subunits in multimeric receptor complexes.

To provide progress in understanding purinergic signaling in the sense of hearing and related diseases, it would be promising to generate, in addition to V60L knock-out mice, mice carrying the two other mutations. For D273Y, we expect similar effects to P2X2 knock-out mice, but the possible suppression of the P2X3-mediated response could improve our knowledge in cells expressing P2X2 and P2X3 subunits. For G353R, it is even more exciting because of the relatively weak effect in heteromeric P2X2/3 channels and the ion dependency. It could be a tool to provide more specific functions of P2X2 channels in physiological and pathological states.

Taken together, studying disease-related mutations contribute to the understanding of important biomolecules from biophysical properties to diseases and this is necessary to pave the way for the development of specific strategies to treat them.

## Data Availability

Data, plasmids and software codes are available upon reasonable request.

## Abbreviations

The following abbreviations are used in this manuscript:

ATP	Adenosine triphosphate
fATP	Fluorescent adenosine triphosphate (2-[DY-547P1]-AHT-ATP)
hP2X2	Human P2X2 receptor
hP2X2/3	Human P2X2/3 receptor
KI	Knock-in
PBS	Phosphate-buffered saline
SEM	Standard error of the mean
SGNs	Spiral ganglion neurons
TM1	Transmembrane domain 1
TM2	Transmembrane domain 2
wt	Wildtype
